# Alzheimer´s Disease in the Perspective of Neuroimmunology

**DOI:** 10.2174/1874205X01812010050

**Published:** 2018-06-29

**Authors:** Ricardo B. Maccioni, Andrea González, Víctor Andrade, Nicole Cortés, José Pablo Tapia, Leonardo Guzmán-Martínez

**Affiliations:** Laboratory of Neuroscience, Faculty of Science, University of Chile & International Center for Biomedicine (ICC), Santiago, Chile

**Keywords:** Alzheimer´s disease, Tau protein, Neuroimmunology, The neuroimmunomodulation theory, Microglial cells, Proinflammatory cytokines

## Abstract

**Background:**

Alzheimer’s Disease (AD) is a severe neurodegenerative disorder that includes the occurrence of behavioral disorders as well as memory and cognitive impairment as major symptoms. AD affects around 12% of the aged population in the world. Considerable research efforts have pointed to the role of innate immunity as the main culprit in the pathogenesis of AD. In this context, and according to with our neuroimmunomodulation theory, microglial activation modifies the cross-talks between microglia and neurons. We postulated that glial activation triggered by “damage signals” activates a pathological molecular cascade that finally leads to hyperphosphorylation and oligomerization of the tau protein. Interestingly, these modifications correlate with the gradual cognitive impairment of patients with the AD. Microglial activation is determined by the nature and strength of the stimulus. In the AD, a continuous activation state of microglia appears to generate neuronal injury and neurodegeneration, producing the outflow of pathological tau from the inner of neurons to the extraneuronal space. Released tau, together with the contribution of ApoE4 protein, would then produce reactivation of microglia, thus inducing a positive feedback that stimulates the vicious cycle in neurodegeneration.

**Conclusion:**

Nevertheless, from the pathophysiological perspective AD is significantly more than a loss of memory. In the initial stages of AD pathogenesis, variations in the dopaminergic pathway along with serotonin diminution play an important role. This may explain why depression is associated with the onset of AD. All these pathophysiological events take place together with immunomodulatory changes that trigger tau oligomerization in the course of neurofibrillary tangles formation. Interestingly, mood disorders appear to be followed by neuroinflammatory processes and structural/functional alterations that lead to cognitive impairment in the context of AD.

## 1 THE UNIFIED HYPOTHESIS OF NEUROIMMUNOMODULATION

Several hypotheses have been postulated to explain the pathogenesis of AD, a multifactorial disease. There are many triggering signals that can promote the onset of the disease, independently, or by an association of several of them. From the histopathological point of view, AD exhibits two main features: the formation of Senile Plaques (SP) formed by progressive pathological assemblies of the Aβ peptide, and the Neurofibrillary Tangles (NFT) formed by the assembly of the hyperphosphorylated variant of the microtubule-associated protein tau. Tau is particularly important because its self-polymerization under pathological conditions, causes the death of neurons and their connections, preventing brain regions from communicating with each other. In the majority of cases, tau pathology first appears in the memory areas of the brain, known as the entorhinal cortex and the hippocampal formation [1]. This has been shown to occur many years before patients have any symptoms of the disease.

It is known that both the amyloid peptide and tau protein have the tendency to self-associate into polymeric structures, due to anomalous conformation derived from the content of β-pleated sheet structure. This implies that the lack of appropriate folding of these polypeptides leads to the formation of self-assembled supramolecular structures such as SP and NFT. In this context, AD is considered a protein conformational disease. Self-assemblies normally occurs in biological systems such as microtubules and microfilaments, and also in the organization of viruses [2]. However several pathological associations are found not only in the SP and NFT, but also in synuclein proteins of the Parkinson`s disease, huntingtin in Hungtinton´s disease, and several other examples [3]. In this context, the amyloid hypothesis has been postulated, based on the idea that amyloid aggregation is the critical event in AD pathogenesis [4], as well the tau hypothesis in that tau modifications is the main culprit of the disease [5]. As a matter of fact, both hypotheses share part of the apparently real situation. The amyloid peptide is one of the factors that promote tau hyperphosphorylation as demonstrated in our laboratory using cellular models [6] and also by other investigators [7, 8]. Moreover, both hypotheses have cumulated growing evidence from *in vitro* and animal studies, but clearly, tau hypothesis agrees with the clinical observations in patients [9]. It is worth to point out that current search for novel drugs to control AD is progressively expanding from investigations based on tau as the main target [10].

**Fig. (1):**
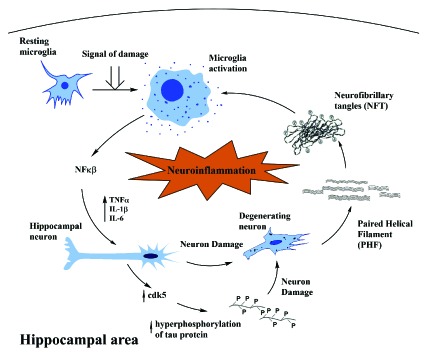
Schematic representation of the signaling cascade for neuronal degeneration, on the basis of the neuroimmunomodulation theory (12). Activation of microglia is followed by a release of pro-inflammatory mediators that finally promotes tau hypephosphorylation and its aggregation into PHF. Tau release from degenerated neurons triggers microglial reactivation, thus generating a vicious cycle in the pathway to degeneration of neuronal cells in pathological processes like Alzheimer’s disease and tauopathies.

We postulated the neuroimmunomodulation hypothesis [11-13], which indicates that the beginning of AD is mainly an outcome of the reaction of microglia to “damage signals” or tau oligomers (Fig. **1**). These elicit a neuro-inflammatory response, inducing an anomalous cascade of signaling with the release of the Nuclear Factor Kappa β (NFkB), overproduction of proinflammatory cytokines and the consequent activation of their neuronal receptors. This activation promotes an augmentation in the expression of the protein kinase protein kinase CDK5 and the formation of a stable complex with p35 [14]. In addition, an activation of the GSK3β (glycogen synthase kinase 3 beta) simultaneously occurs. Overactive protein kinases trigger tau hyperphosphorylation and the subsequent self-aggregation linked with neuronal degeneration [15, 16]. Increasing evidence suggests that tau oligomers and polymers released from neurodegenerated neurons can re-activate microglial cells, thus promoting a positive feedback mechanism associated with the continuous cascade of altered molecular signaling responsible for neuronal degeneration. This neuroimmune mechanism occurs in tauopathies and AD [17]. Tau filaments and PHFs released from degenerating neurons seem to trigger re-activation of microglial cells (Fig. **1**).

These molecular alterations appear to be associated with the erroneous misfolding of tau, a molecular event that causes the collapse of the cytoskeleton [18]. In this context, the neuroinflammatory hypothesis appears to be a more general postulate involved in several tauopathies of a neurodegenerative nature [19, 20]. More interesting, there is a link between different behavioral disorders and anxiety with the activation of neuroinflammatory processes, and they appear to be involved in the pathway in the AD progression [21].

## 2 NEUROINFLAMMATION

Based on the central concept of “damage signals” described above, the definition of neuroinflammation corresponds to the reaction of the central nervous system to exogenous and/or endogenous factors that interfere with the normal cellular homeostasis. This inflammatory response is frequently activated from a traumatic or infection episode. Nonetheless, this reaction has been defined as one of the main axes in the development of neurodegeneration. Possibly, throughout this mechanism, there is a significant death of neurons, in contrast with the initial impairment [22]. The preceding phenomena appear to be implicated in every neurological disorder, as well as developmental pathologies. Inflammation has a principal role in prompting diverse neurodegenerative diseases, such as AD, Parkinson’s and amyotrophic lateral sclerosis, among others (20). In the AD, a persistent inflammatory state is likely to pioneer neural injury, subsequently, neuronal cells death, which thus promote the secretion of pathological forms of tau protein to the extra neuronal environment. It has been demonstrated that some tau oligomers have the capacity of activated microglial cells. Moreover, previous reports have shown that tau oligomers can activate microglia., and in this regard, they afterward activate a positive feedback mechanism, producing a persistent injury to neuronal cells [12, 23, 24]. Furthermore, an important overexpression of inflammatory intermediaries has been described near to Aβ and the paired helical filaments PHF, in the AD, which is related to vastly affected brain regions in the neurodegenerative pathology [13]. Other factors contribute to neuroinflammation but in a silent way, and corresponds to chronic metabolic diseases such as diabetes, hypertension, traumatic lesions in the brain or clinical depression [16]. In a similar way, stroke and atherosclerosis have been also considered risk factors, producing injury or even death in the central nervous system (CNS) tissue. Throughout natural aging, there is a basal chronic activation of pro-inflammatory factors in similar areas, promoting to an even higher susceptibility to neuropsychiatric disorders [25]. If we project our view of neuropsychiatric disorders, clinical depression and anxiety symptoms have been correlated with IL-6, IL-8, TNF alpha, C-reactive protein and adipokines [16, 26]. Finally, the continuous immune response of the CNS, point to the continuous production of neurotrophic factors by the microglia [12].

The over-activation of the immune response in the CNS compromise the generation of neurotrophic factors and the release of cytotoxic agents to the microglial cells [12]. Microglial cells are extensively spread in every region of the CNS, particularly in the hippocampus and the substantia nigra [27]. In this context, we can suggest that the effect of this positive feedback that trigger microglial activation can give us new insights into the genesis and progression in neurodegenerative diseases.

## 3 MICROGLIAL CELLS ARE DETERMINANT FOR THE NEUROINFLAMMATORY PROCESS

Now we can focus on the structural and functional aspects of the microglial cells that seem to be relevant for the neuroinflammatory phenomena. Microglia presents an irregular morphology with a larger nucleus, representing between 5 to 20% of the total glial cell population in the CNS. They have the capacity to produce phagocytosis and release of cytotoxic factors, working as an antigen presentation cell [28]. Microglial cells derive from macrophages formed during the hematopoietic stages in the primitive yolk sac [29] and migrating to the neural tube during development [30]. Their physiological roles are pivotal for maintaining the normal homeostasis in the CNS, even under pathological conditions [13]. They have the capacity of recognizing diverse damage signals which could be considered as a probable injury for the CNS. Part of this pathological signaling mechanism involves the action of i) microorganisms, ii) abnormal endogenous proteins, iii) complement factors, iv) antibodies, and v) cytokines, chemokines, among others. These harmful mediators are recognized by receptors such as toll-like ones (TLR), triggering the activation state of microglia [27, 31]. Under the normal circumstances, microglia regulates the expression of diverse surface markers, for example, the major histocompatibility complex II (CMH-II) and pattern of molecular recognition receptors (PRRs). After these interplay, the release of proinflammatory cytokines is activated. These include interleukin I beta (IL-1β), interleukin 6 (IL-6), Interleukin 12 (IL-12), Interferon Gamma (IFN-γ) and the Tumor Necrosis Factor Alpha (TNF-α) (13). In the meantime, there is synthesis and release of cytotoxic factors with a low biological half-life, such as superoxide radicals (O_2_^-^), Nitric Oxide (NO) and other Reactive Oxygen Species (ROS) [32, 33].

Throughout the different stages of brain development, microglia plays a definite role in apoptotic cells abolition. On the other hand, in the cerebellum, microglial cells control phagocytosis of Purkinje neurons next to cell death mediated by caspase-3. Consequently, microglial cells are involved in synapse removal throughout brain development after birth [34]. Lastly, the course of its activation will be related to the context, intensity and of the type of stimulus produced. According to these features, the microglial cells might generate a neuroprotector or neuroinflammatory effect. Thus, a balance between neurotoxicity and neuroprotection determines the microglial functional consequence of neurological diseases or/and specific brain disorder [13]. As mentioned above, microglia mediates the immune response in the central nervous system CNS, To achieve this mission, the microglia turns into a functional polarized stage, with the characteristic of accomplishing a precise effector program.

Microglia displays two polarized forms, one of them develop the traditional proinflammatory response, which is the most usual phenotype. Another form causes an anti-inflammatory effect focused to restore a damaged region by an acute injury [35]. In addition, microglial cells express numerous receptors in the membrane surface and, participates in the release of several soluble factors. The stimulated cell, with the pro-inflammatory phenotype, regulates factors such as CD16, CD32, CD64, CD86, IL-1b, IL-6, IL-12, IL-23 and TNF-α. Modulation of the inducible nitric oxide synthase (iNOS) and chemokines is also attained. Anti-inflammatory phenotypes also regulate positively arginase-1 (Arg-1), the manose receptor (CD206) as well as Insulin Growth Factor-1 (IGF- 1). Interestingly, all these different proteins factors and receptors, promote the activation of microglia to finally results in the production of additional cytokines and inflammatory factors which seems to be the cause of apoptotic processes of neuronal cells in several neurodegenerative pathologies [20]. Microorganisms and their related secreted proteins such as LPS and others are identified by the the Toll receptors family. Thus apoptotic neurons are recognized by diverse receptor systems acting as mediators, such as asialoglycoproteins, vitronectin, and phosphatidylserine [36]. Several reports have demonstrated that after microglial activation, there is an overexpression of numerous receptors and ligands that belong to the main chemokines families (CC, CXC, and CX3C). Some of these mediators are similarly found in astrocytes, suggesting that such mediators are impor5tant as signals in microglia communication phenomena. In this context, it has been postulated that CX3CR1 and its ligand, fraktaline (CX3CL1), which are also present in neurons, have a main role in neuronal signaling with the microglial cells [37]. There are various factors that regulate the phagocytic function of microglial cells. Another component is the Chloride Intracellular Channel (CLIC1). Through its pharmacological inhibition or by the negative regulation by an interference RNA, these factors disturb the normal phagocytic activity of the microglia. Instead, there is evidence that the Ciliary Neurotrophic Factor (CNTF) triggers phagocytosis events by using a Ca^2+^ -mediated mechanism [38]. In summary, microglia can be modulated by several external factors or by endogenous peptides and proteins, which prompts an over activated state, thus secreting inflammatory molecules, as well as ROS and reactive nitrogen species. These damaging molecules will, in turn, evoke toxicity near the neuronal population [39].

## CONCLUSION

Resting microglia is vulnerable to diverse damage signals including different oligomeric forms of tau protein, thus leading to activation of microglial cells. Subsequently, a set of molecules with an inflammatory activity appear to be released into the extracellular media, promoting neuronal damage and degeneration. In essence, different tau molecular species are released from the interior of neurons onto the extracellular space. Those species produce a positive feedback in the inflammatory cycle, prompting neuronal degeneration in pathologies such as AD. The nutraceutical compound Brain Up-10 displays an anti-aggregation effect on tau oligomers, decreasing damage signals. In the meantime, it boosts neuritogenesis. Tau assembly appears to be an appropriate target for the treatment of AD.
